# Novel Extended Tetraether Lipids Found in a High‐CO_2_ Geyser

**DOI:** 10.1111/1462-2920.70286

**Published:** 2026-03-20

**Authors:** Janina Groninga, Leonie Wittig, Feriel Bouderka, Till L. V. Bornemann, Julius S. Lipp, Florence Schubotz, Saskia Keden, Alexander J. Probst, Kai‐Uwe Hinrichs

**Affiliations:** ^1^ MARUM ‐ Center for Marine Environmental Sciences University of Bremen Bremen Germany; ^2^ Faculty of Geosciences University of Bremen Bremen Germany; ^3^ Environmental Metagenomics, Research Center One Health Ruhr of the University Alliance Ruhr University of Duisburg‐Essen Essen Germany; ^4^ Centre for Water and Environmental Research (ZWU) University of Duisburg‐Essen Essen Germany

**Keywords:** archaea, cold‐water geyser, iGDGTs, membrane lipids, UHPLC–MS

## Abstract

The growing research into the archaeal lipidome has uncovered a remarkable structural diversity in isoprenoidal glycerol dialkyl glycerol tetraethers (iGDGTs) and revealed complex membrane adaptations, especially in extreme environments. We performed a comprehensive analysis of the lipidome from the subsurface aquifer of the CO_2_‐rich, cold‐water Geyser Andernach (Germany), using ultra‐high‐resolution mass spectrometry. We detected iGDGT‐0, presumably derived from the dominant community member *Candidatus* Altiarchaeum, providing supporting evidence for its ability to synthesise tetraethers, as previously predicted from metagenomic data. Beyond the typical iGDGT‐0 and acyclic glycerol trialkyl glycerol tetraether (iGTGT‐0), we discovered novel structural derivatives, here referred to as extended iGDGTs and iGTGTs, characterised by the asymmetrical addition of up to two isoprenoid units to only one of their hydrocarbon side chains, analogous to those found in extended archaeols. The apparent absence of GDGT ring synthase A and B genes in the corresponding metagenome‐assembled genome raises the possibility that the producing archaea may utilise extended iGDGTs as a membrane adaptation to cope with the nutrient‐depleted conditions of the geyser environment, highlighting the adaptive flexibility of archaea to extreme physicochemical conditions.

## Graphical abstract

Characterisation of the archaeal lipidome of the CO_2_‐rich subsurface aquifer of the Geyser Andernach reveals novel extended tetraether lipids analogous to extended archaeols, likely produced by *Candidatus* Altiarchaeum, and potentially functioning as a membrane adaptation strategy to nutrient‐deprived conditions.

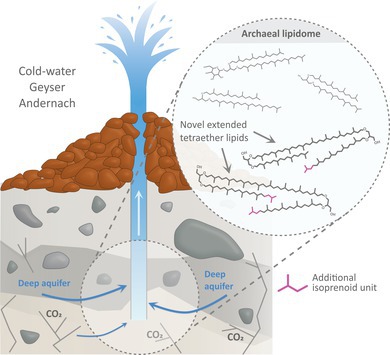

## 1 Introduction

Archaeal cell membranes differ fundamentally from those found in Bacteria or Eukaryotes, and harbour distinct biophysical properties that ensure cellular integrity even under hostile environmental conditions. This resilience is grounded in the molecular structure of their lipids, which comprise exclusively ether‐linked isoprenoid chains attached to a glycerol‐1‐phosphate (G1P) backbone (Koga and Morii 2007; Koga 2012). Broadly, archaeal membrane lipids can be categorised into two main types: diether lipids, such as archaeols (ARs), which form conventional bilayer membranes, and tetraether lipids (isoprenoidal glycerol dialkyl glycerol tetraethers; iGDGTs), which build membrane‐spanning monolayers (De Rosa and Gambacorta 1988; Valentine 2007).

Since the first description of iGDGTs in the 1970s by Langworthy (Langworthy 1977), the research on tetraether lipids has continued to grow, and over the subsequent decades, numerous structural modifications have been reported. These include the incorporation of up to eight cyclopentane rings (introduced via GDGT ring synthases (Grs) (Zeng et al. 2022)), and one cyclohexane ring in addition to four cyclopentane moieties in the *Nitrososphaerota*‐specific crenarchaeol lipid (Sinninghe Damsté et al. 2002). Other structural iGDGT modifications include methylated derivatives in hyperthermophilic archaea (Knappy 2010; Garcia et al. 2024), hydroxylated iGDGTs, which have been proposed to act as temperature proxies (Liu, Lipp, et al. 2012; Huguet et al. 2013, 2017), or unsaturated iGDGTs (Zhu et al. 2014). Cross‐linked iGDGTs, known as glycerol monoalkyl glycerol tetraethers (iGMGTs), in which the biphytanyl chains are connected via a covalent carbon–carbon bridge, represent another specific and unusual modification limited to thermophilic archaea such as *Methanothermus sociabilis* and *Ignisphaera aggregans* (Morii et al. 1998; Knappy et al. 2011; Garcia et al. 2024). More recently, another unusual class of tetraether lipids, known as butanetriol dialkyl glycerol tetraethers (BDGTs) and pentanetriol glycerol tetraethers (PDGTs), which are characterised by a butanetriol or pentanetriol backbone, have been described (Becker et al. 2016; Coffinet et al. 2020). Initially, BDGTs were linked to the methanogen *Methanomassiliicoccus luminyensis* (Becker et al. 2016; Coffinet et al. 2020) but new evidence shows that Bathyarchaeia, a highly abundant archaeal class, are capable of BDGT synthesis as well (Dong et al. 2025). Accordingly, the diversity found in tetraether lipids not only reflects adaptations to various external conditions but can also provide valuable chemotaxonomic indicators for different archaeal communities (Elling et al. 2015, 2017; Jensen et al. 2015; Feyhl‐Buska et al. 2016; Zhou et al. 2020).

Similar modifications have been reported for AR, the diether precursors of membrane‐spanning iGDGTs (Lloyd et al. 2022; Zeng et al. 2022). Besides the typical AR with two C_20_‐chains (2,3‐di‐*O*‐phytanyl‐*sn*‐glycerol), extended variants that include one or two C_25_‐chains (2‐*O*‐sesterterpanyl‐3‐*O*‐phytanyl‐*sn*‐glycerol or 2,3‐di‐*O*‐sesterterpanyl‐*sn*‐glycerol) have been described as another class of archaeal lipids that form membranes suited for strong osmotic stress and high salinity (De Rosa et al. 1982; Vandier et al. 2021). Traditionally, these extended ARs (ext‐ARs) were attributed to halophilic archaea inhabiting various hypersaline environments (De Rosa et al. 1982; Teixidor et al. 1993; Dawson et al. 2012; Birgel et al. 2014); however, recent findings support a more widespread occurrence than previously assumed (Becker et al. 2016; Natalicchio et al. 2017; Vandier et al. 2021). For instance, ext‐AR and its intact derivatives, such as diglycosidic (2G) ext‐AR, were identified in Crystal Geyser, a CO_2_‐driven, cold‐water geyser in Utah, USA (Probst et al. 2020), and in biofilms from the sulfidic Mühlbacher Schwefelquelle (Regensburg, Germany) (Probst et al. 2014; Perras et al. 2015). Notably, these extended AR lipids have been attributed to the anaerobic, and CO_2_‐dependent archaeon *Candidatus* Altiarchaeum of the order *Ca*. Altiarchaeales (also referred to as clade Alti‐1, Bird et al. 2016) within the phylum Altiarchaeota, which represents the most abundant population in these aforementioned subsurface environments (Henneberger et al. 2006; Probst et al. 2014, 2018; Bornemann et al. 2022). Similar geochemical conditions likely promote the dominance of *Ca*. Altiarchaeum at our study site, the cold‐water Geyser Andernach (GA), Germany (Bornemann et al. 2022), whose lipid inventory, thus far, remains unexplored.

Low‐energy environments, such as the deep subsurface of the GA (Bornemann et al. 2022), often drive unique adaptations of cellular membranes and thus provide an ideal setting for discovering novel or unusual lipid structures. In this study, we describe yet another novel modification of iGDGTs, characterised by extended isoprenoidal side chains, analogous to those found in extended ARs. These unusual extended iGDGTs not only expand the known diversity of archaeal lipids but also provide new insight into potential alternative membrane adaptation strategies likely employed by *Ca*. Altiarchaeum in a deep subsurface environment.

## 2 Material and Methods

### 2.1 Study Site and Sampling

Subsurface water samples were collected from the world's highest erupting cold‐water geyser that is situated in the Middle Rhine Valley near Koblenz in western Germany (50.448588° N, 7.375355° E) and was first drilled in 1903 (Bräuer et al. 2013; Dittrich 2019). The GA reaches a temperature of around 18°C and has a total depth of 351.5 m, granting access to a shale‐hosted aquifer for groundwater supply, with the upper 83 m of the borehole annulus being sealed with cement to prevent any surface water contamination (Dittrich 2019; Bornemann et al. 2022). The up to 60 m high eruptions are fueled by geological degassing of mainly CO_2_ of magmatic origin and traces of H_2_ and H_2_S beneath the Eifel region and can be controlled with mechanical shutters (Bräuer et al. 2013; Barros et al. 2020). The concentrations of dissolved CO_2_ and HCO_3_^−^ can reach up to 1500 mg L^−1^ and 5700 mg L^−1^, respectively (Bornemann et al. 2022). These high‐CO_2_ subsurface waters support a mesophilic microbial population dominated by *Ca*. Altiarchaeum (Bornemann et al. 2022).

Samples for lipid analysis were retrieved by collecting biomass from subsurface water onto glass fibre filters with a pore size of 0.7 μm (Ø = 142 mm; Whatman, 1825142) in June 2025, using an installed tap on the side of the geyser to prevent surface contamination and without the GA erupting. The water was filled into sterile containers and afterwards pumped through teflon tubes for filtration. The filters were immediately stored on dry ice on‐site and then moved to −18°C in the laboratory until further processing. On each filter, biomass from ~80 to 140 L of subsurface water was collected. Additional material from the GA used in this study stems from sampling conducted for Bornemann et al. (2022) and has been collected for metagenomic analyses as well as lipid analysis as described therein. For these samples, water was collected in sterile containers during the eruption and subsequently filtered onto 0.1 μm polytetrafluoroethylene (PTFE) filters (Ø = 142 mm; Merck Millipore, JVWP14225).

### 2.2 Sample Preparation and Extraction

Total lipid extracts (TLEs) from the filters were obtained using the modified Bligh & Dyer protocol (Sturt et al. 2004). According to the protocol, the lipids were extracted in four steps using monopotassium phosphate buffer (8.7 g KH_2_PO_4_ per L; pH 7.4) for the first two extraction steps and a trichloroacetic acid buffer (50 g L^−1^; pH 2) for the following two extraction steps. Following each extraction, the samples were centrifuged for 10 min at 1250 rpm, after which the supernatant was decanted into a separatory funnel. Equal volumes of dichloromethane (DCM) and MilliQ water were added to induce phase separation. The aqueous phase was extracted three times with DCM, after which the combined organic phases were collected and subsequently washed three times using MilliQ water. The collected organic phases were evaporated under a gentle stream of N_2_ and the dry TLEs were stored at −18°C until further processing.

### 2.3 UHPLC‐ESI‐timsTOF‐MS^2^

Archaeal lipids were analysed using a Dionex Ultimate 3000RS ultra‐high‐performance liquid chromatography (UHPLC) system (Thermo Fisher Scientific, Bremen, Germany) connected to a trapped ion mobility time‐of‐flight mass spectrometer (timsTOF HT‐MS), equipped with a vacuum‐insulated probe‐heated electrospray ionisation (VIP‐HESI) ion source in positive mode (Bruker Daltonics, Bremen, Germany). A reversed phase (RP) Acquity UPLC BEH C_18_ column (1.7 μm; 2.1 × 150 mm, Waters Corporation, Eschborn, Germany) fitted with a pre‐column was used for chromatographic separation. The solvent system consisted of eluent A (MeOH:H_2_O (85:15) + 0.04% (v:v) HCO_2_H and 0.1% (v:v) NH_3(aq)_) as well as eluent B (MeOH:IPA (1:1) + 0.04% (v:v) HCO_2_H and 0.1% (v:v) NH_3(aq)_) and was used after the following two methods:

*Method A*: Following a method by Wörmer et al. (2013), lipids were separated at a column temperature of 65°C and with a flow rate of 0.4 mL min^−1^ using the following gradient: initial conditions were at 0% eluent B and 100% eluent A, increasing to 15% eluent B by 2 min, 85% eluent B by 20 min, and reaching 100% eluent B by 28 min. The column was re‐equilibrated at 0% eluent B until 34 min.

*Method B*: The separation was performed using a method based on Hopmans et al. (2021) with a column temperature of 30°C, a flow rate of 0.2 mL min^−1^ and following a modified gradient (Meyer et al. 2025): starting with 5% eluent B and 95% eluent A for 3 min, eluent B was increased to 60% by 12 min, followed by a further increase to 100% eluent B over the next 38 min (up to 50 min total). The gradient was maintained until 80 min, followed by a decrease to 5% eluent B over 1 min, which was then held until the end of the gradient at 90 min. This method is used to improve the chromatographic separation of high‐mass molecules such as iGDGTs, if necessary.

Samples were dissolved in MeOH:DCM (9:1, v:v). Each measurement was started with a loop injection of 20 μL of calibration mix (10 mM sodium formate cluster solution:Agilent ESI‐L tuning mix (1:4, v:v), Agilent Technologies, Santa Clara, USA) to enable an internal calibration of mass and ion mobility accuracy. The mass accuracy was better than 1 ppm and the mobility calibration was < 0.2%.

The ESI *m/z* scan ranged from 50 to 3000 with positive ionisation mode, the drying gas flow was set at 8.0 L min^−1^, drying gas temperature at 230°C, nebuliser gas pressure at 4.0 bar, sheath gas at 300°C, and sheath gas flow at 3 L min^−1^. Capillary voltage was set to 4500 V and end plate offset to 500 V. MS/MS stepping was applied to improve detection in the low‐ and high‐mass ranges. For MS/MS analysis, a mobility‐dependent collision energy table with two collision energies that were combined to one scan was used in parallel accumulation serial fragmentation (PASEF) mode with two ramps and a target intensity of 60,000 and active exclusion for 6 s. The inverse mobility 1/K_0_ scan ranged from 0.80 to 2.30 Vs cm^−2^ with a 100 ms ramp time.

DataAnalysis 6.2 (Bruker Daltonics, Bremen, Germany) was used for the evaluation of ESI‐timsTOF and APCI‐QTOF data. Lipids were identified based on their exact masses, retention times, and diagnostic MS^2^ fragmentation patterns (Tables S1 and S2; Figures S1–S3). Individual ion chromatograms were extracted with a mass window of ±0.01 Da, and semi‐quantification was performed relative to an injection standard (2 ng GTGT‐C_46_ per injection, Huguet et al. 2006) added to the extract prior to the measurement. All reported lipid concentrations must be considered semi‐quantitative as standards for each individual lipid species were not available. To account for differences in ionisation efficiency among the various lipid species, we calculated response factors based on calibration curves of standard solutions containing structurally similar compounds, including iGDGT‐0, monoglycosidic iGDGT‐0 (1G‐iGDGT‐0), AR, and diglycosidic AR (2G‐AR). Table S2 indicates which standard has been used for the quantification of which archaeal lipids.

### 2.4 UHPLC‐APCI‐QTOF‐MS^2^

Measurements with atmospheric‐pressure chemical ionisation (APCI) were performed using a Dionex Ultimate 3000RS ultra‐high‐performance liquid chromatography (UHPLC) system (Thermo Fisher Scientific, Bremen, Germany) coupled to a maXis plus ultra‐high‐resolution quadrupole time‐of‐flight mass spectrometer (QTOF‐MS) in positive APCI mode (Bruker Daltonics, Bremen, Germany).

Two normal‐phase Acquity UPLC BEH Amide columns (1.7 μm; 2.1 × 150 mm, Waters Corporation, Eschborn, Germany) fitted with a pre‐column were used following the method by Becker et al. (2013). The flow rate was kept at 0.5 mL min^−1^ with a column temperature of 50°C. The following gradient was used for eluent A (*n*‐hexane) and eluent B (*n*‐hexane:IPA (9:1, v:v)):

Starting with 3% eluent B and 97% eluent A for the first 2 min, eluent B was increased to 5% and used until 10 min total. Afterwards, eluent B was increased to 10% for 10 min (up to 20 min total) and then further increased to 20% for 15 min (up to 35 min total). 50% of eluent B was used for the next 10 min (up to 45 min total), followed by 100% eluent B for 6 min (up to 51 min total). The column was equilibrated at 3% eluent B for 9 min (up to 60 min total).

Samples were dissolved in *n*‐hexane:IPA (99.5:0.5, v:v). Each measurement was terminated with the injection of a calibration mix (ESI‐L tuning mix, Agilent Technologies, Santa Clara, USA) to allow mass calibration. Each mass spectrum was lock mass‐calibrated with a lock mass compound (922.0098 Da), which was added in the ion source. The mass accuracy was typically better than 1 ppm.

The APCI source parameters were as follows: scan *m/z* range from 50 to 2000, drying gas flow 6.0 L min^−1^, drying gas temperature 180°C, nebuliser gas pressure 2.0 bar, and collision energy 6 eV.

### 2.5 Detection of iGDGT Synthesis Marker Genes in Geyser Andernach Metagenomes and Reconstruction of Their Evolutionary History

To constrain the potential producers of iGDGTs and their extended tetraether derivatives in Geyser Andernach, we screened the metagenome assembly for homology of key marker genes involved in tetraether and iGDGT cycloalkyl ring biosynthesis. Reference sequences of tetraether lipid synthesis genes (*tes*) were obtained from Zeng et al. (2022), totaling 1601 sequences. For the GDGT ring synthase genes (*grs*), we included the *grsA* and *grsB* sequences identified in Zeng et al. (2019), all other archaeal homologues of these genes reported in that same study, and additional homologous *grsA* sequences obtained from the InterPro database for a total of 61 sequences (Blum et al. 2025). These reference datasets were used as queries to search for homologous sequences in the Geyser Andernach metagenomic assemblies. The assemblies were generated, as described in Bornemann et al. (2022), from environmental samples analogous to those used for lipid analysis. Homology searches were performed using DIAMOND BLASTp (Buchfink et al. 2015, v2.1.13.167) with a minimum 50% amino acid identity, 50% alignment coverage, and an E‐value cutoff of 1e^−5^. When present, corresponding hits were searched in the metagenome‐assembled genomes (MAGs) generated from the same dataset, previously reconstructed in Bornemann et al. (2022). The identified MAG taxonomy was assigned according to the Genome Taxonomy Database (GTDB, Release R10‐RS226) (Parks et al. 2025).

To reconstruct the phylogeny of the *tes* genes recovered from the GA metagenome, reference sequences from Zeng et al. (2022) were included to provide context. Recovered sequences were aligned alongside these references using MAFFT (Katoh et al. 2002, v7.407), and the resulting alignment was trimmed using TrimAl (Capella‐Gutiérrez et al. 2009, v1.5.rev0). Maximum‐likelihood phylogenetic trees were reconstructed from the trimmed alignment using IQ‐TREE with the LG + C20 + G + F model (Nguyen et al. 2015, v 3.0.1) and the resulting trees were visualised and annotated using iTOL (Letunic and Bork 2021).

## 3 Results

### 3.1 Archaeal Lipid Composition in the Geyser Andernach

**FIGURE 1:**
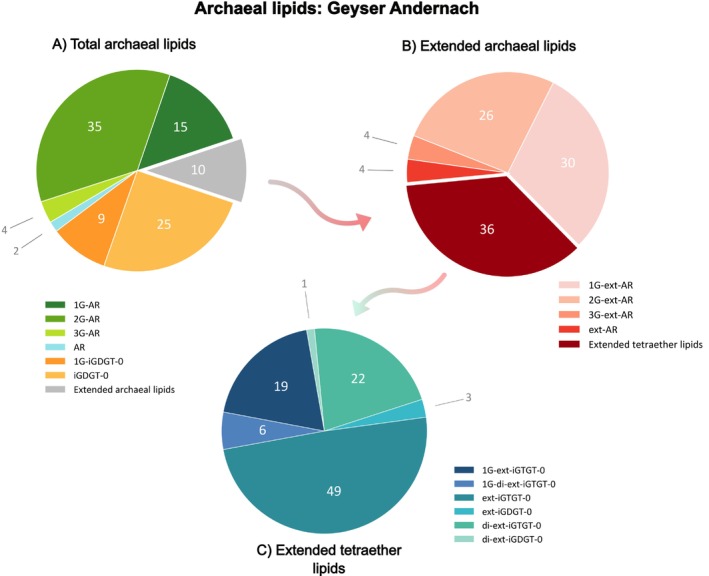
Relative abundances (%) of archaeal lipids detected in the erupting water from the Geyser Andernach (Germany), analysed with reversed‐phase UHPLC‐ESI‐timsTOF using method A. Corresponding lipid concentrations in ng/extract can be found in Tables S1 and S2. The left chart (A) shows the relative abundance of the total archaeal lipid inventory, with the grey sector showing lipids comprised of extended C_25_‐sesterterpanyl chains. A breakdown of the extended archaeal lipid fraction, including novel compounds tentatively identified as extended iGTGTs and extended iGDGTs (c.f. 3.2), is featured in the right pie chart (B). A further breakdown of specifically extended tetraether lipids is shown in panel C.

The investigation of the GA lipidome revealed a major proportion of glycosidic archaeols with up to three glucose moieties (Figure 1A, Tables S1 and S2). Within the total archaeal lipid pool, 2G‐AR (35%), followed by 1G‐AR (15%), stood out as the most abundant lipid species. Additionally, tetraether lipids, specifically iGDGT‐0 and 1G‐iGDGT‐0, were detected in considerable proportions, accounting for roughly 34% of the archaeal lipidome (Figure 1A). Furthermore, approximately 10% of the archaeal lipid inventory consisted of lipids featuring extended versions of the usual phytanyl and biphytanyl moieties. Ext‐ARs with either a 1G or 2G head group dominate this fraction and together represent more than half of the extended archaeal lipid pool (Figure 1B). Most remarkable was the detection of several unusual tetraether lipids, tentatively identified as extended or di‐extended iGDGTs and iGTGTs (Figure 1C), which appear to comprise additional isoprenoid units, analogous to those found in ext‐AR. Interestingly, extended iGTGT‐0 (ext‐iGTGT‐0; 1.8% of total lipid pool) and di‐extended iGTGT‐0 (di‐ext‐iGTGT‐0; 0.8% of total lipid pool) are more abundant than their extended iGDGT analogs, which together only account for 0.1% of the total lipid pool. This trend is also evident in their glycosidic forms, as 1G‐ext‐iGTGT‐0 and 1G‐di‐ext‐iGTGT‐0 were detected, whereas intact extended iGDGT remained below the detection limit. In total, the extended tetraether lipids account for approximately 10.6% of the total tetraether lipid pool (Tables S1 and S2).

### 3.2 Structural Characterisation of Extended iGTGTs and iGDGTs

**FIGURE 2:**
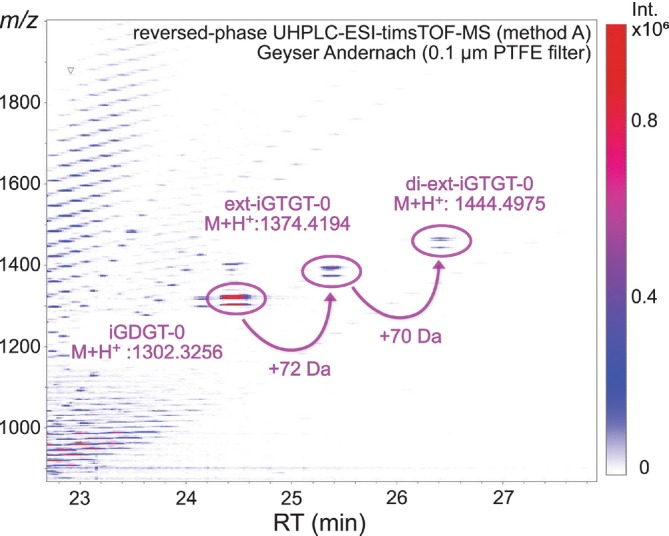
Heatmap of a positive RP‐UHPLC‐ESI‐timsTOF‐MS measurement (method A) of Geyser Andernach filter extracts, highlighting iGDGT‐0, ext‐iGTGT‐0, and di‐ext‐iGTGT‐0. Red colour indicates higher signal intensities shown on a logarithmic scale.

Reversed phase UHPLC‐ESI‐timsTOF‐MS revealed a distinct series of isoprenoidal tetraether lipids with retention times between 24 and 27 min, starting with iGDGT‐0 (24.4 min) and followed by two lipids tentatively annotated as ext‐iGTGT‐0 (25.3 min) and di‐ext‐iGTGT‐0 (26.3 min) (Figure 2). In addition to the ~1‐min shift in retention time, the two extended compounds exhibited a stepwise mass increase, with a first increment of +72 Da relative to iGDGT‐0, reflecting one additional isoprenoid unit as well as two hydrogen atoms due to the tri‐alkyl structure of iGTGTs. The second increment of +70 Da corresponds to the addition of yet another isoprenoid unit within the iGTGT lipid structure. Due to the close elution pattern of iGDGTs and iGTGTs, we performed additional normal‐phase APCI‐QTOF‐MS^2^ analysis to facilitate better chromatographic separation and enable structural characterisation based on the MS^2^ fragmentation pattern.

**FIGURE 3:**
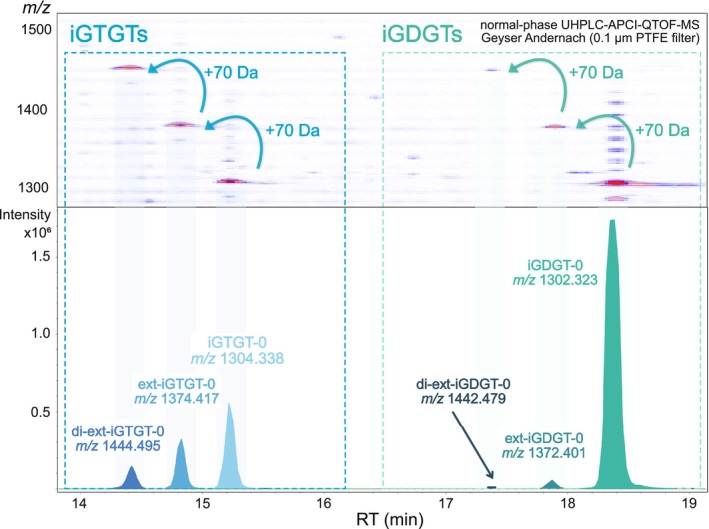
Heatmaps and extracted ion chromatograms illustrating the series of regular and extended iGTGTs and iGDGTs in UHPLC‐APCI‐QTOF‐MS analysis in extracts from the subsurface waters of the Geyser Andernach. Red colouring in the density map indicates high signal intensities equivalent to those shown in the extracted ion chromatograms below.

UHPLC‐APCI‐QTOF‐MS^2^ not only confirmed the distinct series of iGTGTs but also revealed a corresponding iGDGT series that was barely distinguishable in RP‐UHPLC‐ESI‐timsTOF‐MS. Starting with iGTGT‐0 ([C_86_H_174_O_6_ + H]^+^: observed *m/z*: 1304.339; theoretical *m/z*: 1304.338) and iGDGT‐0 ([C_86_H_172_O_6_ + H]^+^: observed *m/z*: 1302.326; theoretical *m/z*: 1302.323), respectively, each series is characterised by a mass increase of ~70 Da (C_5_H_10_), consistent with the successive addition of isoprenoid units (Figure 3). Next to the extended iGDGTs and iGTGTs, traces (< 1.2% of the archaeal lipidome) of cycloalkylated iGDGTs (iGDGT‐1 to iGDGT‐4) and glycerol monoalkyl glycerol tetraether (GMGT) were detected in the APCI‐based measurements only (Table S3).

**FIGURE 4:**
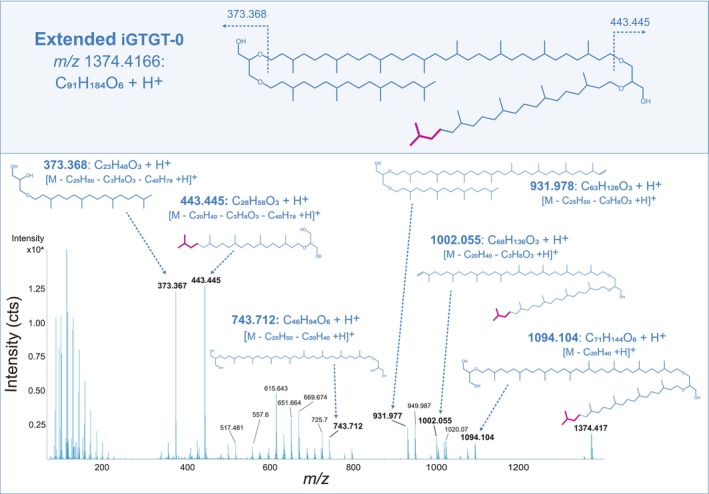
Proposed chemical structure of an extended iGTGT‐0 (C_91_H_184_O_6_) found in lipid extracts from the eruption waters of the Geyser Andernach (upper panel). UHPLC‐APCI‐QTOF‐MS^2^ spectrum of extended iGTGT‐0 [M + H]^+^, emphasising diagnostic MS^2^ fragment ions and the corresponding annotated neutral losses (lower panel). Magenta colouring indicates the additional isoprenoid unit characteristic for the extended iGTGT‐0 structure, which can be traced throughout the MS^2^ fragmentation pattern.

UHPLC‐APCI‐MS^2^ spectra of *m/z* 1374.416 support the presence of one extended side chain featuring an additional isoprenoid unit, as evidenced by a recurring mass increase of 70 Da throughout the MS^2^ spectrum when compared to the regular iGTGT‐0 (Figure 4). Most prominent is the fragment ion at *m/z* 443.445 (C_28_H_58_O_3_) reflecting a C_25_‐sesterterpanyl side chain attached to glycerol, alongside the regular C_20_‐phytanyl chain attached to glycerol (*m/z* 373.367; C_23_H_48_O_3_) (Becker et al. 2016). The characteristic +70 Da isoprenoid extension can be traced across several higher‐mass ions, most prominently at *m/z* 1002.055 ([M‐C_23_H_48_O_3_ + H]^+^) and *m/z* 1094.104 ([M‐C_20_H_40_ + H]^+^), reflecting the successive neutral loss of phytane (C_20_H_40_) and one glycerol unit (C_3_H_8_O_3_). The consistent occurrence of extended C_25_ chain fragments alongside those characteristic of the typical C_20_‐phytanyl chain (e.g., *m/z* 373.368, *m/z* 931.978, *m/z* 949.987), and C_40_ chain fragments (e.g., *m/z* 743.712, *m/z* 651.665, *m/z* 615.644) support a general structure consistent with an extended iGTGT‐0 (Figures 4 and S1).

**FIGURE 5:**
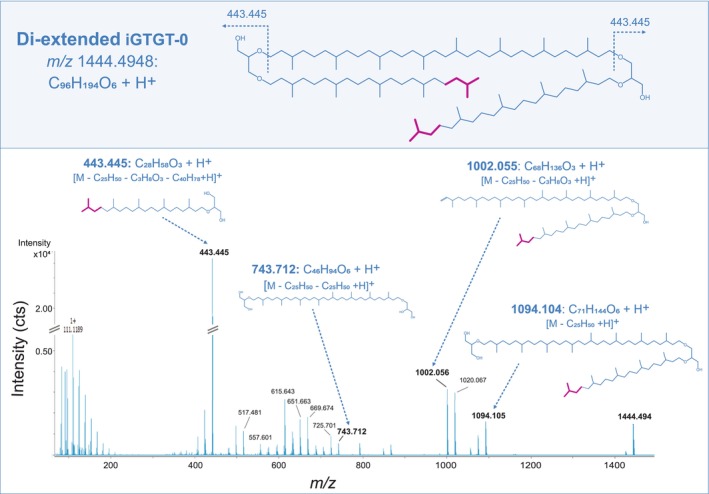
Proposed chemical structure of di‐extended iGTGT‐0 (C_96_H_194_O_6_) found in lipid extracts from the eruption waters of the Geyser Andernach (upper panel). UHPLC‐APCI‐QTOF‐MS^2^ spectrum of di‐extended iGTGT‐0 [M + H]^+^ with characteristic MS^2^ fragment ions and the corresponding annotated neutral losses (lower panel). Magenta colouring indicates two additional isoprenoid units within the di‐extended iGTGT‐0 structure.

The earliest eluting compound of this series with *m/z* 1444.495 is consistent with the molecular formula [C_96_H_194_O_6_ + H]^+^ (theoretical *m/z* 1444.495). Relative to iGTGT‐0 (C_86_H_174_O_6_; *m/z* 1304.338), this represents a mass increase of 140.156 Da, indicating the incorporation of two additional isoprenoid units (C_10_H_20_). The MS^2^ spectrum has the glycerol‐C_25_‐sesterterpanyl fragment (*m/z* 443.445) as base peak and lacks the characteristic glycerol‐C_20_‐phytanyl fragment ion at *m/z* 373.367. Fragment ions indicative of a C_40_ chain (e.g., *m/z* 743.712, *m/z* 651.665, *m/z* 615.644), and high intensity peaks at *m/z* 1094.105, *m/z* 1002.067, and *m/z* 443.445 corroborate an iGTGT‐0 with two extended C_25_ side chains (di‐ext‐iGTGT‐0) (Figures 5 and S2).

Eluting roughly 3 min later, we observed an additional series of compounds, encompassing. *m/z* 1302.324, corresponding to iGDGT‐0, *m/z* 1372.406, and *m/z* 1442.488, featuring the same successive addition of a +70 Da isoprenoid motif indicative of the novel extended iGDGTs.

**FIGURE 6:**
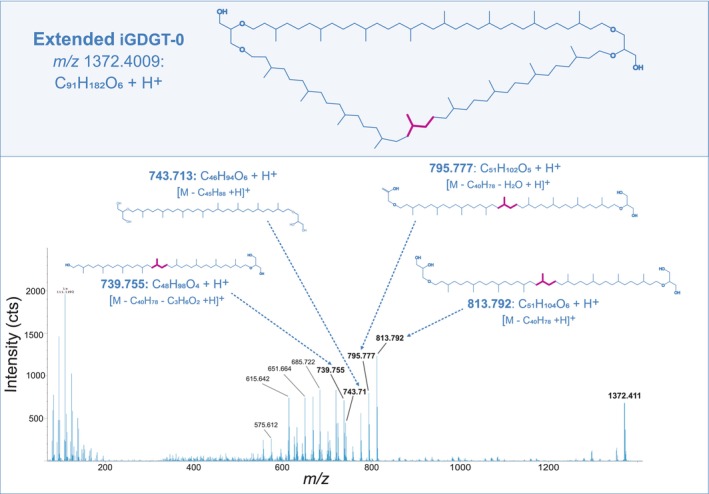
Proposed chemical structure of extended iGDGT‐0 (C_91_H_182_O_6_) found in lipid extracts from the eruption waters of the Geyser Andernach (upper panel). UHPLC‐APCI‐QTOF‐MS^2^ spectrum of extended iGDGT‐0 [M + H]^+^, emphasising diagnostic MS^2^ fragment ions and the corresponding annotated neutral losses (lower panel). Magenta colouring highlights the additional isoprenoid unit characteristic of the extended iGDGT‐0 structure, which is found throughout the MS^2^ fragmentation pattern.

MS^2^ spectra of *m/z* 1372.406 ([C_91_H_182_O_6_ + H]^+^; theoretical *m/z* 1372.401) confirmed this hypothesis as we observed a high intensity fragment ion at *m/z* 813.792 reflecting a C_45_ side chain linked at both ends to a glycerol moiety, resulting from the loss of one C_40_‐biphytanyl chain ([M‐C_40_H_78_ + H]^+^) (Figures 6 and S3). Congruently, the fragment ions *m/z* 743.713, *m/z* 651.664, and *m/z* 615.642 typically observed in the iGDGT core structure result from the neutral loss of one C_45_‐nonaprenyl chain ([M‐C_45_H_88_ + H]^+^). This distinct fragmentation pattern is evidence of the presence of asymmetrical iGDGTs that harbour one extended side chain. The distinct elution pattern in APCI‐QTOF‐MS and +70 Da mass increase alluded to additional occurrence of a di‐extended iGDGT presumably comprising one C_50_‐decaprenyl chain. Due to the low abundance, no MS^2^ spectrum was available to further support the tentative structural assignment; however, we interpret *m/z* 1444.445 to contain one C_40_‐biphytanyl chain and one extended C_50_‐decaprenyl chain as opposed to two C_45_‐nonaprenyl chains, due to the structural trend observed in the di‐extended iGTGT, where the C_40_‐biphytanyl chain has been conserved (Figure 5).

**FIGURE 7:**
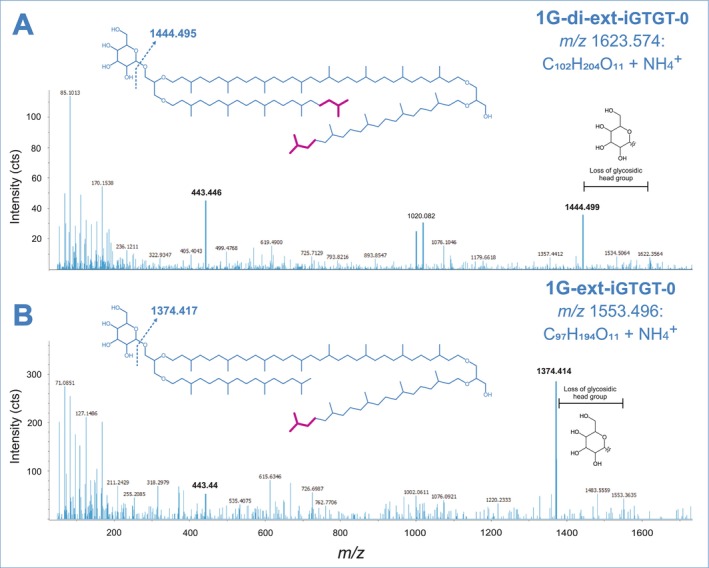
Proposed chemical structures of monoglycosidic extended (C_97_H_194_O_11_) and di‐extended iGTGT‐0 (C_102_H_204_O_11_) found in lipid extracts from the subsurface waters of the Geyser Andernach and corresponding UHPLC‐ESI‐timsTOF‐MS^2^ spectra emphasising diagnostic MS^2^ fragment ions (method B). The major diagnostic ions at *m/z* 1374.414 (B) and *m/z* 1444.499 (A) arise from the neutral loss of the glycosidic head group ([M‐C_6_H_10_O_5_ + H]^+^). Magenta colouring indicates the additional isoprenoid units characteristic of the extended tetraether lipid structures.

Additionally, RP‐timsTOF‐MS^2^ measurements of glass fibre (0.7 μm) filter extracts confirmed the presence of glycosidic extended and di‐extended iGTGTs (1G‐ext‐iGTGT‐0 and 1G‐di‐ext‐iGTGT‐0) at *m/z* 1553.496 and *m/z* 1623.574 (Figure 7). Although these lipids were only detected in trace amounts, MS^2^ spectra revealed a fragment ion at *m/z* 1374.414 and *m/z* 1444.499, respectively, reflecting the neutral loss of the glycosidic head group ([M‐C_6_H_10_O_5_ + H]^+^) and *m/z* 443.4 representing the extended C_25_‐sesterterpanyl side chain. In general, analyses of extracts of glass fibre (0.7 μm) filters and PTFE (0.1 μm) filters resulted in highly similar lipid inventories.

For additional structural information on the C_45_ and C_50_ side chains, gas chromatography (GC)‐MS measurements were attempted. However, the expected C_45_ and C_50_ isoprenoid hydrocarbons, acquired from ether cleavage, reduction, and purification, remained below the detection limit of GC–MS analysis due to their overall low abundance compared to iGDGT‐0. Their extended counterparts only represent a minor fraction of the lipidome, accounting for less than 0.5% relative to iGDGT‐0 (Tables S1 and S2).

### 3.3 Genomic Support for iGDGT Synthesis by *Ca.* Altiarchaeum and a Distinct Evolutionary History of the Corresponding Gene Across the Altiarchaeota Phylum

We identified a single *tes* gene homologue in the Geyser Andernach metagenome assembly using DIAMOND BLASTp (identity > 50%, coverage > 50% and e‐value < 1e^−5^). This gene was assigned to a previously published *Ca*. Altiarchaeum MAG (accession GCA_018260755.1; GTDB, Release R10‐RS226) from the Alti‐1 clade (*Ca*. Altiarchaeales), which was recovered from the same metagenome (Bornemann et al. 2022). The amino acid sequence of the identified tetraether synthase protein (Tes) homologue can be found in Table S4. By contrast, homologues of the GDGT ring synthase genes *grsA* and *grsB* were not detected in the GA metagenome assembly using the same approach.

To recover the evolutionary relationships of the *tes* genes in the Altiarchaeota phylum, we reconstructed a maximum‐likelihood phylogenetic tree, including the *tes* gene from this study and reference *tes* sequences from a previously published dataset (Zeng et al. 2022). Additionally, we included the *tes* gene sequences from the Alti‐2 clade of the Altiarchaeota obtained from Guaymas Basin samples (GCA_003663045.1; GCA_003663165.1; GCA_002254565.1) and White Oak River Basin samples (GCA_001723855.1), in which extended tetraether lipids were detected (Table S5).

## 4 Discussion

The subsurface waters of the GA harbour a distinct microbial population, where *Ca*. Altiarchaeum represents the sole detected archaeal lineage, recruiting for 42.8% of all metagenomic reads (Bornemann et al. 2022). Consequently, we regard *Ca*. Altiarchaeum as the primary and likely exclusive source of the archaeal lipids detected in the geyser waters. The GA archaeal lipidome (Figure 1) is strikingly consistent with the initial lipid characterisation of *Ca*. Altiarchaeum (SM1‐MSI strain, Mühlbacher Schwefelquelle) by Probst et al. (2014), who reported a strong dominance of glycosidic ARs (total 91%), accompanied by minor contributions of glycosidic ext‐ARs. Contrary to our observations in the GA, neither iGDGTs nor extended iGDGTs or iGTGTs have been reported for the biofilms dominated by *Ca*. Altiarchaeum from a cold sulfidic spring near Regensburg by Probst et al. (2014). However, it should be noted that different UHPLC–MS methods were applied that were not specifically targeted toward the detection of core lipids.

The extension of isoprenoid chains in archaeal membrane lipids has already been well documented in archaeal diether lipids (De Rosa et al. 1982; Vandier et al. 2021). In contrast to the ubiquitously distributed archaeol (Tourte et al. 2020), extended archaeol is typically much more constrained and considered a lipid biomarker for halophilic Euryarchaeota, such as *Halobacteriales*, *Haloferacales*, and *Natrialbales* (Dawson et al. 2012) and is often used as an indicator for hypersaline or evaporitic environments (Birgel et al. 2014). However, we note that there are known exceptions, where ext‐ARs have been identified in non‐halophilic methanogens: *Methanomassiliicoccus luminyensis* (Becker et al. 2016) or *Methanosarcina barkerii* (De Rosa et al. 1986), marine lithological sequences with no evidence of hypersalinity (Natalicchio et al. 2017) and multiple reports related to *Ca*. Altiarchaeum (Probst et al. 2014, 2020; Perras et al. 2015), including the cold‐water GA investigated in this study. The most prominent hypothesis explaining the occurrence of ext‐AR in *Haloarchaea* relates to their role in mitigating strong osmotic stress. The incorporation of longer, asymmetric C_20_‐C_25_ AR in a potentially interlocking structure increases membrane thickness and reduces passive ion leakage stress (De Rosa et al. 1982; Vandier et al. 2021), which in turn helps to maintain the proton motive force under extreme salinity. Although the GA water is best described as brackish, it receives no surface inputs of organic matter and is highly limited in phosphate. Together with the absence of light, we hypothesise that its nutrient‐ and energy‐limited character, rather than salinity, may act as a comparable stressor, driving these physiological adaptations (Bornemann et al. 2022).

Our study marks the first report of tetraether lipids attributable to the dominating microbial population of the GA, *Ca*. Altiarchaeum (Bornemann et al. 2022), based on the detection of iGDGT‐0 and its intact counterpart 1G‐iGDGT‐0. Interestingly, a recent genomic survey using Basic Local Alignment Search Tool (BLASTp) already suggested that Altiarchaeota have the potential genetic capacity for iGDGT biosynthesis, as homologues of the tetraether synthase genes (*tes*) have been identified within their genome (Zeng et al. 2022). This radical S‐adenosylmethionine (SAM) enzyme plays a critical role in the formation of iGDGTs by catalysing the coupling of two isoprenoidal diether lipids, primarily archaeols, under the formation of isoprenoid glycerol trialkyl glycerol tetraether (iGTGTs) as intermediates (Koga and Morii 2007; Matsumi et al. 2011; Straub et al. 2018; Lloyd et al. 2022; Zeng et al. 2022). To constrain the likely producers of iGDGTs and extended iGDGTs and to corroborate the biosynthetic potential described by Zeng et al. (2022), we screened the metagenome assembly for a *tes* gene, resulting in a strong match to reference sequences, in a *Ca*. Altiarchaeum (Alti‐1 clade) MAG recovered from GA (Bornemann et al. 2022) (cf. Section 3.3).

Beyond iGDGT‐0, the GA lipidome also features novel iGDGTs and iGTGTs comprising up to two additional isoprenoid units similar to those described in the archaeal C_20_–C_25_ diethers. Given that *Ca*. Altiarchaeum is the dominant prokaryotic and the only archaeal taxon detected via DNA sequencing within the geyser subsurface (Bornemann et al. 2022), and appears to be the primary iGDGT‐producing archae on in this setting, it is most probable that these structurally related tetraether lipids originate from the same source. It is possible that these modified extended iGDGTs and iGTGTs reflect an expanded versatility in the tetraether synthesis in *Ca*. Altiarchaeum, potentially as an adaptation to the unique physicochemical conditions in the deep subsurface environment. We hypothesise that the incorporation of additional isoprenoid units increases the thickness of the cell membrane. Typically, an increased hydrophobic core of the membrane reduces proton and ion permeability, and thus isoprenoid chain extension in tetraether lipids may function in a similar way as previously described for ext‐ARs, which represent a well‐described adaptation strategy to osmotic stress (De Rosa et al. 1982; Vandier et al. 2021). Energy limitation in subsurface environments may represent a comparable stressor that selects for reduced membrane permeability to prevent costly ion and proton leakage and maintain the proton motive force. Overall, a less permeable membrane reduces maintenance energy demands and optimises adenosine triphosphate (ATP) turnover, which may be particularly important considering phosphate limitation (Valentine 2007; Hoehler and Jørgensen 2013; Bornemann et al. 2022). However, the effect of the apparently asymmetric structure of these lipids on membrane packing remains speculative, and it is possible that they may also introduce packing defects, further altering membrane fluidity. To assess how extended tetraether lipids alter membrane properties and influence permeability, membrane modelling and molecular dynamics simulations are required. *In silico* studies, similar to those performed by Huguet et al. (2017), who simulated membranes comprising either iGDGT‐0 or its mono‐hydroxylated variant OH‐iGDGT‐0, may offer further clarity. Additionally, future lipidomic screening of diverse habitats or culturing experiments could aid in constraining the environmental pressures selecting for extended tetraether lipids.

A more common membrane adaptation strategy to environmental stressors, such as temperature, is the incorporation of up to eight cyclopentane rings into the isoprenoid chains (De Rosa and Gambacorta 1988; Lai et al. 2008; Boyd et al. 2011). The insertion of ring moieties increases packing density and rigidifies the lipid membrane, enabling archaea to maintain cell integrity and functionality and prevent ion leakage under extreme temperatures or pH (De Rosa et al. 1980; Gliozzi et al. 1983; Chiu et al. 2023). Considering that *grsA* and *grsB* homologues, which are required for the formation of cyclopentane rings (Zeng et al. 2019), have not been identified in the GA metagenome (cf. Section 3.3), it is probable that *Ca*. Altiarchaeum may not have the biosynthetic capability to produce cyclized iGDGTs. In that regard, the detection of trace amounts of cyclopentane‐bearing iGDGTs in UHPLC‐APCI‐MS analysis seems contradictory (Table S3). Although the absence of *grsA* and *grsB* homologues makes it unlikely that *Ca*. Altiarchaeum are the source of these lipids, it cannot be fully excluded, as the corresponding genes may not have been fully recovered due to MAG fragmentation and incomplete genome reconstruction. Alternatively, it is also conceivable that ring‐bearing iGDGTs originate from another, low‐abundance archaeal taxon that was not captured in the metagenome. Nevertheless, the isoprenoid extension of tetraether lipids is likely attributable to the predominant *Ca*. Altiarchaeum and may represent a novel adaptation strategy to prevent ion leakage in the nutrient‐depleted cold‐water aquifer (Bornemann et al. 2022).

To further strengthen the probable link between extended tetraether lipids and *Ca*. Altiarchaeum, we propose two future research avenues: first, compound‐specific stable carbon isotope analysis of ext‐iGDGTs and ext‐iGTGTs could provide additional support, as lipids associated with *Ca*. Altiarchaeum have been observed to show relatively ^13^C‐depleted isotopic signatures of approximately −71‰ consistent with carbon fixation via the Wood‐Ljungdahl pathway (Probst et al. 2014). Secondly, for an even stronger source attribution, metatranscriptomic analysis to assess whether the tetraether biosynthesis genes are actively expressed in *Ca*. Altiarchaeum under sampling conditions should be performed.

Surprisingly, ext‐iGTGT and di‐ext‐iGTGT were more abundant than their dialkyl iGDGT counterparts. This pattern is also reflected in their glycosylated derivatives, as only 1G‐ext‐iGTGT‐0 and 1G‐di‐ext‐iGTGT‐0 were detected in trace amounts (Figure 1), while intact ext‐iGDGTs remained below the detection limit. iGTGTs are considered a transient intermediate in iGDGT biosynthesis, consistent with their typically low abundance in both environmental samples and cultures (Liu, Summons, and Hinrichs 2012; Bauersachs et al. 2015; Tourte et al. 2022). This may indicate that Tes processes the ext‐AR substrates less efficiently. The larger C_25_‐chain potentially slows the second enzymatic condensation step, creating a kinetic bottleneck and, in turn, causing the accumulation of the extended iGTGT intermediates. However, this interpretation is based on observations from a single site, and more targeted studies are needed to determine whether there are differences in the biosynthesis of regular and extended iGDGTs.

Overall, the close association of these unusual extended tetraether lipids and *Ca*. Altiarchaeum hints that they could potentially serve as a chemotaxonomic indicator for this lineage. However, additional comprehensive lipidomic surveys from sites known to host Altiarchaeota and from cultured representatives are needed to evaluate their true biomarker potential. Alternatively, the occurrence of extended iGDGTs may reflect a more general environmental adaptation to extreme subsurface habitats.

To explore whether these novel ext‐iGDGTs occur beyond the GA subsurface, we performed a preliminary re‐inspection of previously conducted, unpublished lipid datasets from environments including the Guaymas Basin and the White Oak River Basin, known to host Altiarchaeota, particularly of the Alti‐2 clade, based on metagenomic evidence (Bird et al. 2016; Dombrowski et al. 2018; Bornemann et al. 2022) (Table S5). Albeit in traces, we tentatively detected ext‐iGDGT‐0 in the hydrothermally influenced deep‐sea sediment from the Guaymas Basin and di‐ext‐iGDGT‐0 in anoxic, estuarine sediment from the White Oak River Basin, based on the *m/z* and characteristic retention time patterns. It is worth noting that the data outside of GA primarily originate from a retrospective re‐inspection of older UHPLC–MS measurements conducted several years ago (Table S5), when the newly reported extended tetraethers were outside the original analytical focus. Thus, the detection of extended tetraether lipids is qualitative rather than quantitative and should be regarded only as an indication of the occurrence of these lipids at these sites, rather than as an estimate of abundance.

Furthermore, in contrast to GA, where Altiarchaeota dominate, the Guaymas Basin and White Oak River Basin host considerably more diverse archaeal communities, often dominated by Bathyarchaeota, various methanogens, or anaerobic methane‐oxidising archaea (ANME) (Lazar et al. 2015; Ramírez et al. 2020; Teske 2020). This diversity results in a much less straightforward source attribution and therefore, ext‐iGDGTs cannot be confidently linked to Altiarchaeota at those sites. Nevertheless, despite the unresolved archaeal source, their detection still provides a preliminary indication that extended tetraether lipids occur outside of the GA system. Interestingly, the hydrothermal Guaymas Basin sediment and the brackish, anoxic White Oak River Basin sediment represent markedly different environments, and potential environmental drivers for extended tetraether may therefore differ between sites. While in the Guaymas Basin, elevated temperatures are a clear stressor that may favour increased membrane thickness, a comparable driver in the White Oak River sediment is less clearly defined. This uncertainty highlights the need for future systematic screening across diverse habitats to constrain whether the synthesis of these extended lipids indeed reflects specific environmental drivers. Due to their relatively low abundance compared to typical iGDGTs (Figure 1 and Table S5), the extended tetraether lipids may have escaped detection in previous studies and could be more widely distributed than currently recognised. Ultimately, rather than providing concrete evidence for a widespread environmental distribution, these preliminary results should highlight extended tetraether lipids as a promising target for systematic and more targeted lipidomic screening across diverse environmental settings. Future lipidomic investigations employing high‐sensitivity analytical approaches, ideally combined with genomic data, will be needed to resolve the ecological significance as well as their microbial origin across diverse environmental settings.

Given the tentative detection of extended tetraether lipids in additional environments hosting Altiarchaeota, we explored whether the corresponding *tes* sequences from GA, the Guaymas Basin, and the White Oak River Basin may form a distinct phylogenetic clade that could indicate a potential functional specialisation for the utilisation of ext‐AR as substrates. While we did not observe any distinct altiarchaeotal *tes* clade, the phylogenetic tree instead suggests that the altiarchaeotal *tes* genes may have two distinct evolutionary origins: the *tes* sequence representative from the Geyser Andernach Alti‐1 clade clusters with sequences from the Euryarchaeota phylum, whereas the Alti‐2 clade appears more closely related to sequences from the TACK superphylum (Figure S4). However, this analysis is based on a small number of observations of extended tetraether lipids potentially associated with Altiarchaeota, and a more comprehensive phylogenetic analysis of *tes* potentially involved in extended tetraether synthesis will be required for more robust conclusions.

## 5 Conclusion

Our findings add a novel group of isoprenoidal tetraethers to the steadily growing catalogue of archaeal membrane lipids, further underscoring their remarkable structural diversity. The newly detected extended and di‐extended iGTGTs and iGDGTs are characterised by the addition of isoprenoid units, analogous to those described in ext‐AR, while retaining one conventional biphytanyl motif, ultimately resulting in an asymmetrical structure. The dominance of *Ca*. Altiarchaeum in the CO_2_‐rich, nutrient‐depleted subsurface aquifer of the GA, combined with the only *tes* homologue being affiliated with *Ca*. Altiarchaeum strongly implies this lineage as its most probable source. We hypothesise that, at this site, *Ca*. Altiarchaeum may employ the extension of isoprenoidal side chains as a strategy for adapting membrane permeability and energy conservation under nutrient‐ and energy‐limited conditions.

## Author Contributions

**Janina Groninga:** conceptualisation, investigation, formal analysis, visualisation, sample collection, writing – original draft and editing (equal). **Leonie Wittig:** conceptualisation, investigation, formal analysis, sample collection and preparation, methodology, writing – original draft and editing (equal). **Feriel Bouderka:** bioinformatic analysis, processing, sample collection, review and editing. **Till L. V. Bornemann:** sample collection, resources, review and editing. **Julius S. Lipp:** resources, methodology, review and editing. **Florence Schubotz:** conceptualisation, supervision, review and editing. **Saskia Keden:** sample collection, methodology, review and editing. **Alexander J. Probst:** funding acquisition, sample collection, resources, review and editing. **Kai‐Uwe Hinrichs:** conceptualisation, funding acquisition, resources, supervision, review and editing.

## Funding

This work was supported by the European Research Council, 101118631.

## Conflicts of Interest

The authors declare no conflicts of interest.

## Supporting information


**Data S1:** Supporting Information. FASTA file containing the curated set of predicted protein‐coding sequences from *Ca*. Altiarchaeum metagenome‐assembled genome (MAG) GA_180221_E‐1‐1.

**Data S2:** Supporting Information. Functional annotation of the curated predicted genes using eggNOG‐mapper.

**Data S3:** Supporting Information. FASTA file containing the amino acid sequence of the radical SAM tetraether synthase identified in the *Ca.* Altiarchaeum MAG GCA_018260755.1.

**Figure S1:** Proposed fragmentation of ext‐GTGT‐0 [M + H]^+^ based on MS^2^ spectra acquired via APCI‐QTOF‐MS^2^ in positive ionisation mode.
**Figure S2:** Proposed fragmentation of di‐ext‐GTGT‐0 [M + H]^+^ based on MS^2^ spectra acquired via APCI‐QTOF‐MS^2^ in positive ionisation mode.
**Figure S3:** Proposed fragmentation of ext‐GDGT‐0 [M + H]^+^ based on MS^2^ spectra acquired via APCI‐QTOF‐MS^2^ in positive ionisation mode.
**Figure S4:** Maximum‐likelihood phylogenetic tree about the evolutionary relationships of the tes genes, including references from Zeng et al. (2022). Additional archaeal sequences from the Geyser Andernach metagenome of this study, Guaymas Basin (Core 4484 and 4569) and White Oak River Basin are marked in red and resemble sites, in which the tes gene as well as extended tetraether lipids were found.
**Table S1:** Overview of all identified archaeal lipids identified in the erupting water of the Geyser Andernach via UHPLC‐ESI‐timsTOF‐MS. The reported parameters include the chemical formula, monoisotopic mass, three most abundant adducts, retention time (using method A) as well as peak area (arbitrary units), relative abundance, concentration (amount of the respective lipid in ng present in the total lipid extract (ng/TLE)) and inverse reduced ion mobility values of the [M + NH_4_]^+^ adducts. This semi‐quantification was performed relative to an injection standard while accounting for differences in ionisation efficiency using calibration curves of standard solutions (diglycosidic archaeol (2G‐AR), AR, monoglycosidic glycerol dialkyl glycerol tetraether (1G‐iGDGT‐0) and glycerol dialkyl glycerol tetraether (GTGT)‐C_46_ (cf. Section 2.3)). 2G‐AR was used as the standard for intact glycosidic ARs; AR for AR; 1G‐iGDGT‐0 for all intact glycosidic tetraether lipids; and the GTGT‐C_46_ standard for core GDGTs.
**Table S2:** Overview of extended archaeal lipids identified in the erupting water of the Geyser Andernach via UHPLC‐ESI‐timsTOF‐MS. The reported parameters include the chemical formula, monoisotopic mass, three most abundant adducts, retention time (using method A) as well as peak area (arbitrary units), relative abundance, concentration (amount of the respective lipid in ng present in the total lipid extract (ng/TLE)) and inverse reduced ion mobility values of the [M + NH_4_]^+^ adducts. This semi‐quantification was performed relative to an injection standard while accounting for differences in ionisation efficiency using calibration curves of standard solutions (diglycosidic archaeol (2G‐AR), AR, monoglycosidic glycerol dialkyl glycerol tetraether (1G‐iGDGT‐0) and glycerol dialkyl glycerol tetraether (GTGT)‐C_46_ (cf. Section 2.3)). 2G‐AR was used as the standard for intact glycosidic ext‐ARs; AR for extended ARs; 1G‐iGDGT‐0 for all glycosidic ext‐iGTGTs; and the GTGT‐C_46_ standard for extended iGDGTs and iGTGTs.
**Table S3:** Overview of archaeal core lipids obtained via UHPLC‐APCI‐QTOF‐MS measurements of erupting water of the Geyser Andernach, including their chemical formula, the most abundant adduct, *m/z*, retention time as well as peak area in arbitrary units (arb. u.) and relative abundance.
**Table S4:** Amino acid sequence of the tetraether synthase (Tes) homologue identified in the Geyser Andernach metagenome (Bioproject accession no. PRJNA627655) and assigned to the *Ca*. Altiarchaeum MAG (accession GCA_018260755.1).
**Table S5:** Peak areas in arbitrary units (arb. u.) of iGDGT‐0 and its extended analogs (ext‐iGDGT‐0 and di‐ext‐iGDGT‐0) found during retrospective screening of archived lipid extracts and LC–MS data from hydrothermal sediments in the Guaymas Basin and anoxic estuarine sediments of the White Oak River Basin (WOR). Only peaks with an integrated area above 1000 arb. u. were considered. For each sample, the date of the original measurement as well as sampling and measurement information, including ionisation mode (electrospray ionisation (ESI) or atmospheric pressure chemical ionisation (APCI)) and chromatographic separation, are listed.

## Data Availability

All data supporting the findings of this study are available in the Supporting Information (Tables S1–S5). Raw mass spectrometry data are openly available at the Zenodo repository under https://doi.org/10.5281/zenodo.18079306. The Geyser Andernach aquifer metagenome is openly available under the Bioproject accession number PRJNA627655.
