# Supplementary material for: Novel Extended Tetraether Lipids Found in a High‐CO2 Geyser
Source: Environ Microbiol. 2026 Mar 20;28(3):e70286. doi: 10.1111/1462-2920.70286 (PMC13003381; doi:10.1111/1462-2920.70286)
Supplement: Supplementary file 4 — Figure S1: Proposed fragmentation of ext‐GTGT‐0 [M + H]+ based on MS2 spectra acquired via APCI‐QTOF‐MS2 in positive ionisation mode. Figure S2: Proposed fragmentation of di‐ext‐GTGT‐0 [M + H]+ based on MS2 spectra acquired via APCI‐QTOF‐MS2 in positive ionisation mode. Figure S3: Proposed fragmentation of ext‐GDGT‐0 [M + H]+ based on MS2 spectra acquired via APCI‐QTOF‐MS2 in positive ionisation mode. Figure S4: Maximum‐likelihood phylogenetic tree about the evolutionary relationships of the tes genes, including references from Zeng et al. (2022). Additional archaeal sequences from the Geyser Andernach metagenome of this study, Guaymas Basin (Core 4484 and 4569) and White Oak River Basin are marked in red and resemble sites, in which the tes gene as well as extended tetraether lipids were found. Table S1: Overview of all identified archaeal lipids identified in the erupting water of the Geyser Andernach via UHPLC‐ESI‐timsTOF‐MS. The reported parameters include the chemical formula, monoisotopic mass, three most abundant adducts, retention time (using method A) as well as peak area (arbitrary units), relative abundance, concentration (amount of the respective lipid in ng present in the total lipid extract (ng/TLE)) and inverse reduced ion mobility values of the [M + NH4]+ adducts. This semi‐quantification was performed relative to an injection standard while accounting for differences in ionisation efficiency using calibration curves of standard solutions (diglycosidic archaeol (2G‐AR), AR, monoglycosidic glycerol dialkyl glycerol tetraether (1G‐iGDGT‐0) and glycerol dialkyl glycerol tetraether (GTGT)‐C46 (cf. Section 2.3)). 2G‐AR was used as the standard for intact glycosidic ARs; AR for AR; 1G‐iGDGT‐0 for all intact glycosidic tetraether lipids; and the GTGT‐C46 standard for core GDGTs. Table S2: Overview of extended archaeal lipids identified in the erupting water of the Geyser Andernach via UHPLC‐ESI‐timsTOF‐MS. The reported parameters include the chemical formul [file EMI-28-e70286-s002.pdf]

Supplementary Material for:

Novel Extended Tetraether Lipids Found in a High-CO_2 Geyser

Janina Groninga[1*] and Leonie Wittig[1,2*], Feriel Bouderka[3], Till L. V. Bornemann[3,4], Julius S. Lipp[1], Florence Schubotz[1], Saskia Keden[1,2], Alexander J. Probst[3,4], Kai-Uwe Hinrichs[1,2]

[1] MARUM - Center for Marine Environmental Sciences, University of Bremen, Bremen, Germany
[2] Faculty of Geosciences, University of Bremen, Bremen, Germany
[3] Environmental Metagenomics, Research Center One Health Ruhr of the University Alliance Ruhr, University of Duisburg-Essen, Essen, Germany
[4] Centre for Water and Environmental Research (ZWU), University of Duisburg-Essen, Essen, Germany

Corresponding authors: E-mail: jgroninga@marum.de, lwittig@marum.de

*Authors contributed equally

This PDF file includes:

Supplementary Figures S1 to S4

Supplementary Tables S1 to S5

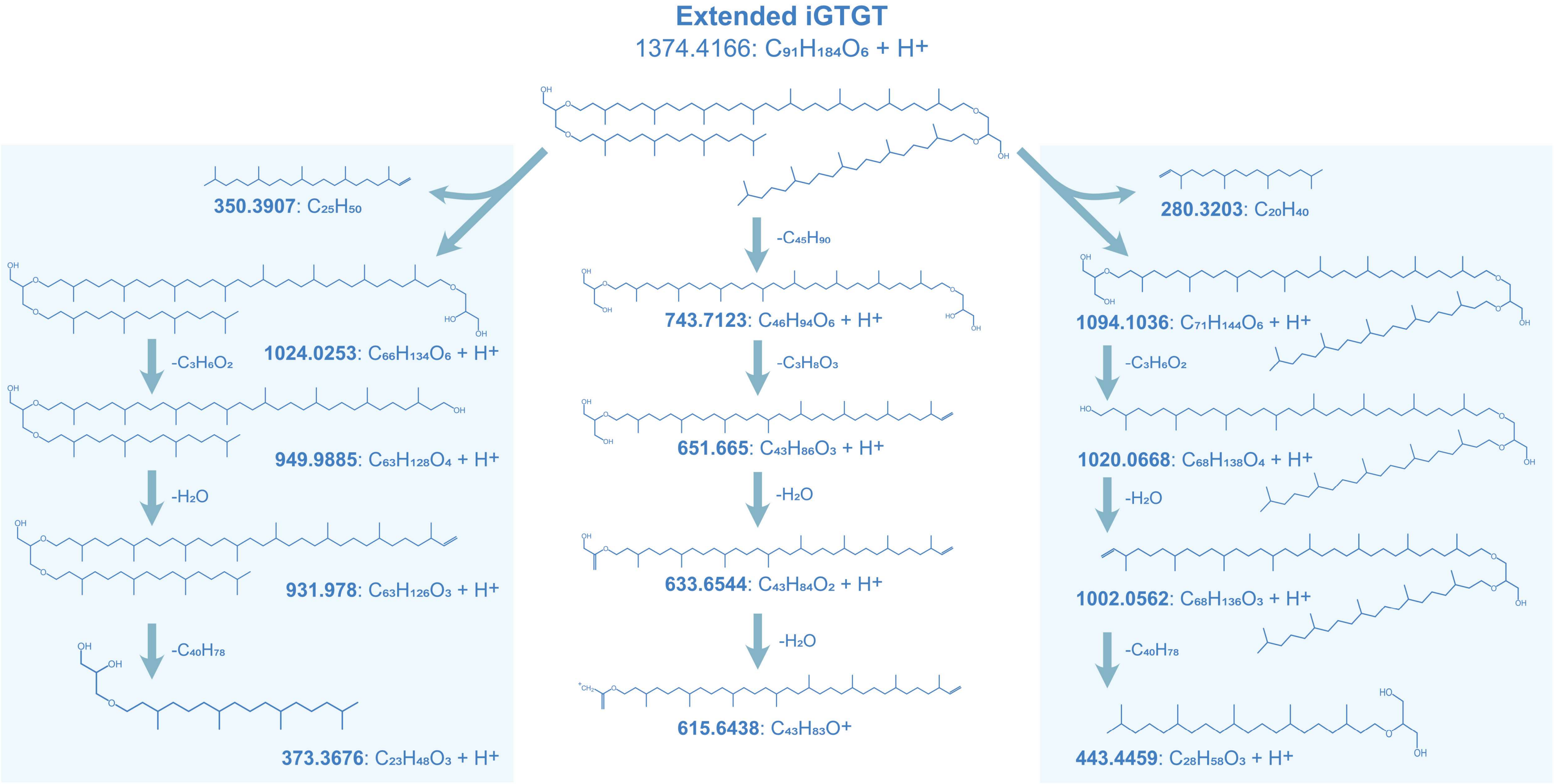

Fig. S1. Proposed fragmentation of ext-GTGT-0 $[M+H]^+$ based on MS^2 spectra acquired via APCI-QTOF-MS^2 in positive ionization mode.

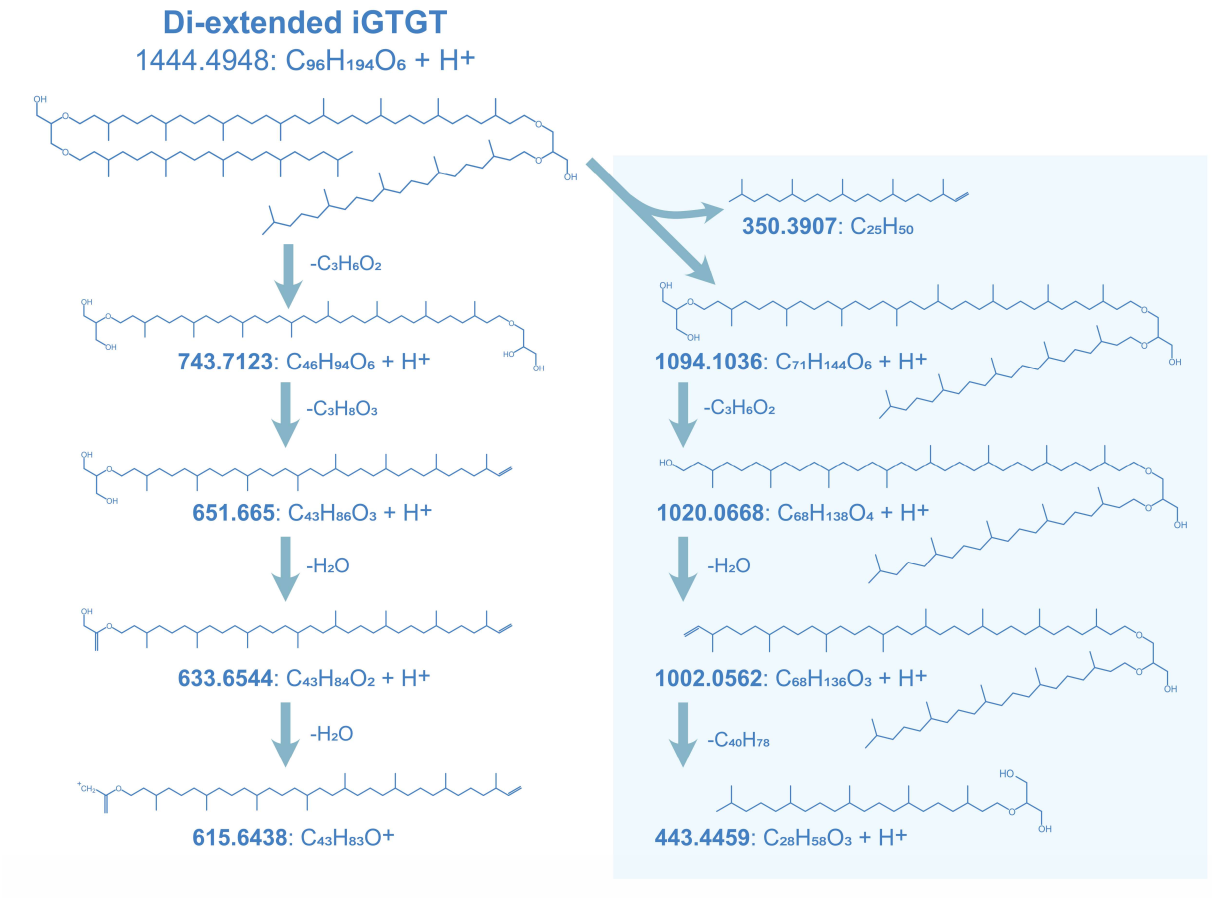

Fig. S2. Proposed fragmentation of di-ext-GTGT-0 $[M+H]^+$ based on MS^2 spectra acquired via APCI-QTOF-MS^2 in positive ionization mode.

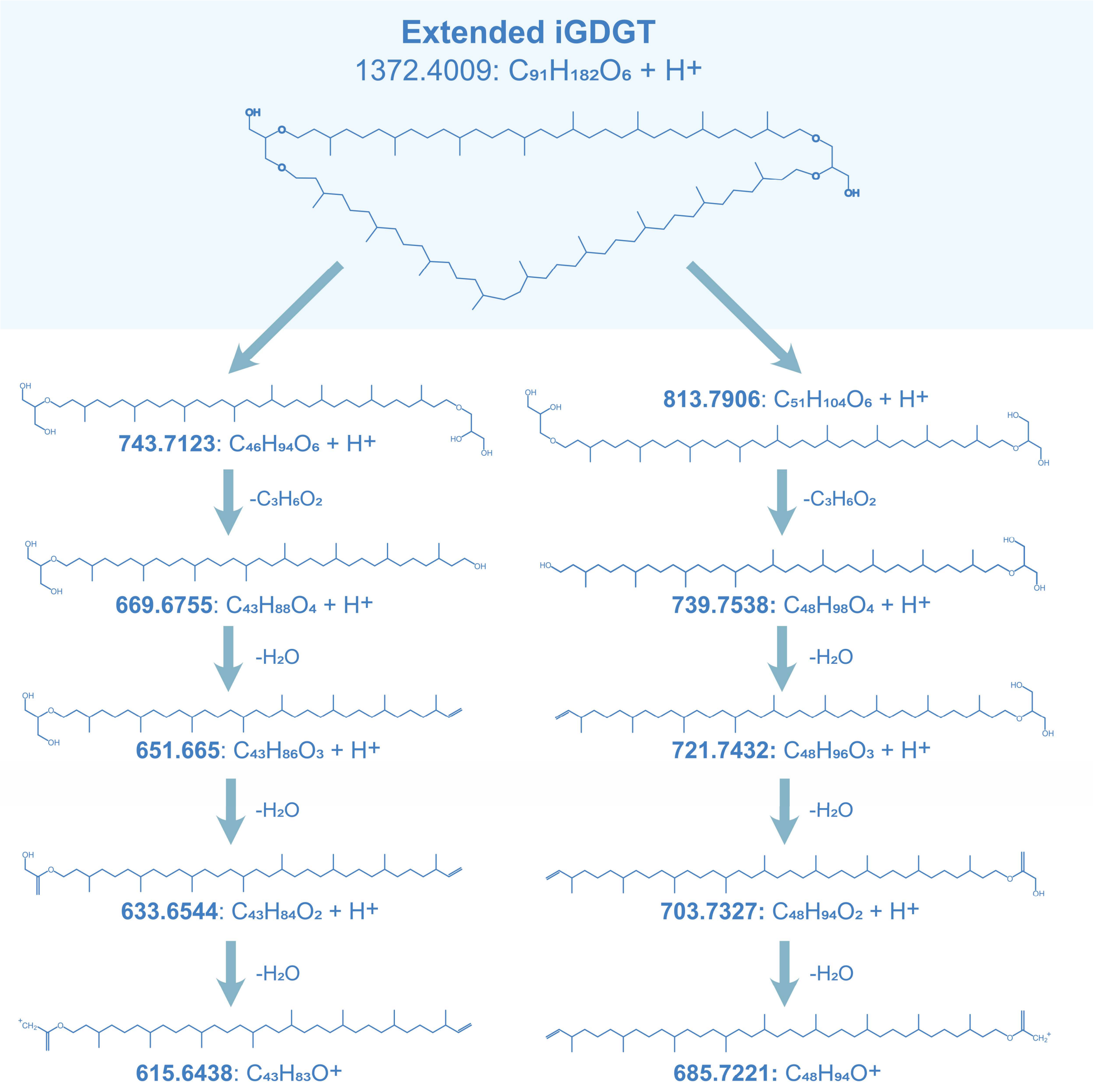

Fig. S3. Proposed fragmentation of ext-GDGT-0 $[M+H]^+$ based on MS^2 spectra acquired via APCI-QTOF-MS^2 in positive ionization mode.

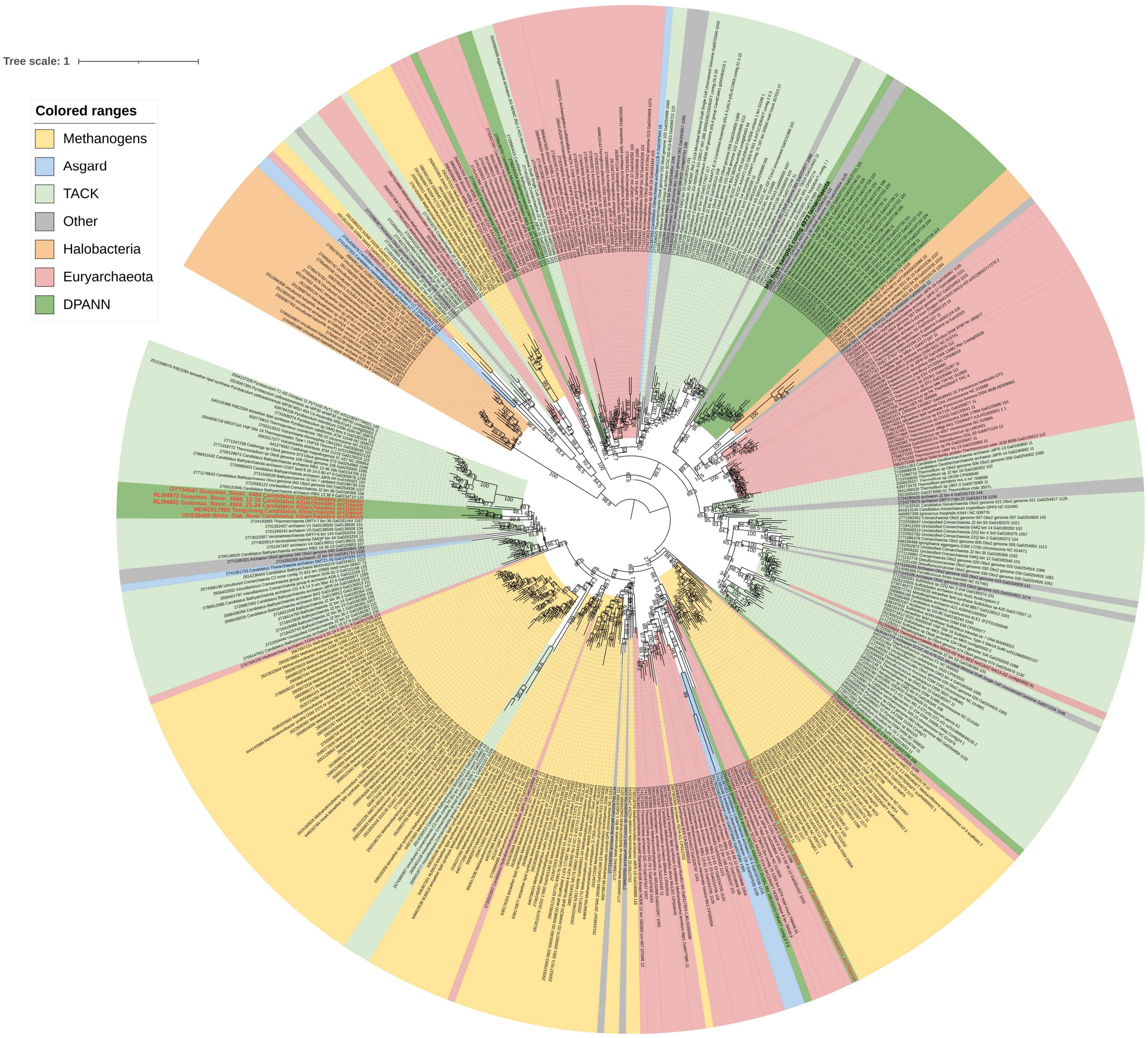

Fig. S4. Maximum-likelihood phylogenetic tree about the evolutionary relationships of the *tes* genes, including references from Zeng et al. (2022). Additional archaeal sequences from the Geyser Andernach metagenome of this study, Guaymas Basin (Core 4484 and 4569) and White Oak River Basin are marked in red and resemble sites, in which the *tes* gene as well as extended tetraether lipids were found.

Table S1. Overview of **all identified archaeal lipids** identified in the erupting water of the Geyser Andernach via UHPLC-ESI-timsTOF-MS. The reported parameters include the chemical formula, monoisotopic mass, three most abundant adducts, retention time (using method A) as well as peak area (arbitrary units), relative abundance, concentration (amount of the respective lipid in ng present in the total lipid extract (ng/TLE)) and inverse reduced ion mobility values of the $[M+NH_4]^+$ adducts. This semi-quantification was performed relative to an injection standard while accounting for differences in ionization efficiency using calibration curves of standard solutions (diglycosidic archaeol (2G-AR), AR, monoglycosidic glycerol dialkyl glycerol tetraether (1G-iGDGT-0) and glycerol dialkyl glycerol tetraether (GTGT)-C46 (cf. section 2.3)). 2G-AR was used as the standard for intact glycosidic ARs; AR for AR; 1G-iGDGT-0 for all intact glycosidic tetraether lipids; and the iGTGT-C_{46} standard for core GDGTs.

Lipid	Chem. formula	Monoisotopic mass	*m/z* Adducts $[M+H]^+$; $[M+NH_4]^+$; $[M+Na]^+$	RT [min]	Peak Area [arb. u.]	Rel. Abundance [%]	Conc. [ng/TLE]	Mobility $1/K_0$ [Vs cm^{-2}]
**1G-AR**	$C_{49}H_{98}O_8$	814.726	815.733; 832.760; 837.715	16.8	7126755	15	6.20	1.53 ± 0.02
**2G-AR**	$C_{55}H_{108}O_{13}$	976.778	977.786; 994.8128; 999.768	15.6	17161294	35	14.93	1.65 ± 0.02
**3G-AR**	$C_{61}H_{118}O_{18}$	1138.381	1139.839; 1156.866; 1161.821	14.8	1725292	4	1.50	1.74 ± 0.02
**AR**	$C_{43}H_{88}O_3$	652.673	653.681; 670.707; 675.663	18.6	4354194	2	0.72	1.38 ± 0.02
**1G-iGDGT-0**	$C_{92}H_{182}O_{11}$	1463.368	1464.375; 1481.402; 1486.357	23.5	2432768	9	3.99	2.06 ± 0.02
**iGDGT-0**	$C_{86}H_{172}O_6$	1301.315	1302.323; 1319.349; 1324.305	24.4	53530108	25	10.69	1.98 ± 0.02
**Extended Lipids**	-	-	-	-	-	10	-	-

Table S2. Overview of **extended archaeal lipids** identified in the erupting water of the Geyser Andernach via UHPLC-ESI-timsTOF-MS. The reported parameters include the chemical formula, monoisotopic mass, three most abundant adducts, retention time (using method A) as well as peak area (arbitrary units), relative abundance, concentration (amount of the respective lipid in ng present in the total lipid extract (ng/TLE)) and inverse reduced ion mobility values of the $[M+NH_4]^+$ adducts. This semi-quantification was performed relative to an injection standard while accounting for differences in ionization efficiency using calibration curves of standard solutions (diglycosidic archaeol (2G-AR), AR, monoglycosidic glycerol dialkyl glycerol tetraether (1G-iGDGT-0) and glycerol dialkyl glycerol tetraether (GTGT)-C_{46} (cf. section 2.3)). 2G-AR was used as the standard for intact glycosidic ext-ARs; AR for extended ARs; 1G-iGDGT-0 for all glycosidic ext-iGTGTs; and the GTGT-C_{46} standard for extended iGDGTs and iGTGTs.

Lipid	Chem. formula	Monoisotopic mass	Adducts $[M+H]^+$; $[M+NH_4]^+$; $[M+Na]^+$	RT [min]	Peak Area [arb. u.]	Rel. Abundance [%]	Conc. [ng/TLE]	Mobility $1/K_0$ [Vs cm^{-2}]
**1G-ext-AR**	$C_{54}H_{108}O_8$	884.804	885.812; 902.838; 907.794	19.0	1511827	30	1.32	1.61 ± 0.02
**2G-ext-AR**	$C_{60}H_{118}O_{13}$	1046.857	1047.864; 1064.891; 1069.846	17.9	1320736	26	1.15	1.71 ± 0.02
**3G-ext-AR**	$C_{66}H_{128}O_{18}$	1208.909	1209.917; 1226.944; 1231.899	17.3	191498	4	0.17	-
**ext-AR**	$C_{48}H_{98}O_3$	722.751	723.759; 740.785; 745.741	20.7	980529	4	0.16	1.46 ± 0.02
**ext-iGTGT-0**	$C_{91}H_{184}O_6$	1373.409	1374.416; 1391.443; 1396.398	25.3	3849205	18	0.77	2.09 ± 0.02
**di-ext-iGTGT-0**	$C_{96}H_{194}O_6$	1443.487	1444.495; 1461.521; 1466.477	26.3	1683153	8	0.34	2.15 ± 0.02
**1G-ext-iGTGT-0**	$C_{97}H_{194}O_{11}$	1535.461	1536.469; 1553.496; 1558.451	24.2	183218	7	0.30	2.17 ± 0.02
**1G-di-ext-iGTGT-0**	$C_{102}H_{204}O_{11}$	1605.540	1606.548; 1623.574; 1628.529	24.9	55766	2	0.09	2.23 ± 0.02
**ext-iGDGT-0**	$C_{91}H_{182}O_6$	1371.393	1372.401; 1389.427; 1394.383	25.1	221317	1	0.04	2.08 ± 0.02
**di-ext-iGDGT-0**	$C_{96}H_{192}O_6$	1441.472	1442.479; 1459.506; 1464.461	26.0	97050	0.4	0.02	2.14 ± 0.02

Table S3. Overview of **archaeal core lipids** obtained via UHPLC-APCI-QTOF-MS measurements of erupting water of the Geyser Andernach, including their chemical formula, the most abundant adduct, *m/z*, retention time as well as peak area in arbitrary units (arb. u.) and relative abundance.

Lipid	Chem. Formula	Adducts	*m/z*	RT [min]	Peak Area [arb. u.]	Rel. Abundance [%]
**di-ext-iGTGT-0**	$C_{96}H_{194}O_6$	$[M+H]^+$	1444.4948	14.4	777716	3.7
**di-ext-iGDGT-0**	$C_{96}H_{192}O_6$	$[M+H]^+$	1442.4792	17.4	18963	0.1
**ext-iGTGT-0**	$C_{91}H_{184}O_6$	$[M+H]^+$	1374.4166	14.8	1689421	8.1
**ext-iGDGT-0**	$C_{91}H_{182}O_6$	$[M+H]^+$	1372.4009	17.9	270903	1.3
**iGDGT-0**	$C_{86}H_{172}O_6$	$[M+H]^+$	1302.3227	18.4	14473976	69.5
**iGTGT-0**	$C_{86}H_{174}O_6$	$[M+H]^+$	1304.3383	15.2	2847365	13.7
**iGDGT-1**	$C_{86}H_{170}O_6$	$[M+H]^+$	1300.3070	18.6	254546	1.2
**iGDGT-1 (Isomer)**	$C_{86}H_{170}O_6$	$[M+H]^+$	1300.3070	18.9	73570	0.4
**iGDGT-2**	$C_{86}H_{168}O_6$	$[M+H]^+$	1298.2914	19.5	82204	0.4
**iGDGT-3**	$C_{86}H_{166}O_6$	$[M+H]^+$	1296.2757	20.2	42081	0.2
**iGDGT-4**	$C_{86}H_{164}O_6$	$[M+H]^+$	1294.2601	20.9	12988	0.1
**iGMGT-0**	$C_{86}H_{170}O_6$	$[M+H]^+$	1300.3070	22.2	219307	1.1
**iGMGT-1**	$C_{86}H_{168}O_6$	$[M+H]^+$	1298.2914	22.8	72509	0.3

Table S4. Amino acid sequence of the tetraether synthase (Tes) homolog identified in the Geyser Andernach metagenome (Bioproject accession no. PRJNA627655) and assigned to the *Ca*. Altiarchaeum MAG (accession GCA_018260755.1)

Accession	Name	Length	Amino acid sequence
Ca. Altiarchaeum MAG (GCA_018260755.1)	Tetraether synthase (Tes)	559 aa	MSQSKVRSRNSAIRNQHSTIMKGLPKETQSLCPECGKIVQARVFERDGKALIEKECPEHG<br>RFSDVYWSDAAMYLKAEKFAYDGQGVSNPRMPVTDGCPFDCGLCDEHLTHTLLANIDLTN<br>RCNLRCPICFANANASGYVYEPDFKTVVGMMRVLRGNRPVPAAAVQFSGGEPTVRPDFLE<br>ILKTASKMGFRHIQVATNGLKMAEDPAFVQKMVNTGLNTAYLQFDGLDDEVYMKLRGRRL<br>LAVKKKAIENCRKSTLSTVLVPTIVKTVNDNQIGAIFDFAIQNSDVVRGINYQPIAFTGR<br>ISMKERERQRFTIPDVCAAIEKHTNGVIKRSDFYPVPVVAPISELVASVAGKPQITFTTH<br>PHCGMATYIFVEKSQKGASDAGGAHIGGGRDNNPKIIPITQFVDVEGFMSDVMQLAARIR<br>GSRFARMRLLLNSGKVLRHIDQKKGPAGLDVKAMIRSVLFNDYQALIKFSWNSLLVGVMH<br>FQDSYNYDIERVKRCGIHQVTPDGRLIPFCAYNGGPTYRTEIERRFGIPLKDFKEANSAG<br>EVGTAEKRRRQPCKTIKSR

Table S5. Peak areas in arbitrary units (arb. u.) of iGDGT-0 and its extended analogs (ext-iGDGT-0 and di-ext-iGDGT-0) found during retrospective screening of archived lipid extracts and LC-MS data from hydrothermal sediments in the Guaymas Basin and anoxic estuarine sediments of the White Oak River Basin (WOR). Only peaks with an integrated area above 1000 arb. u. were considered. For each sample, the date of the original measurement as well as sampling and measurement information, including ionization mode (electrospray ionization (ESI) or atmospheric pressure chemical ionization (APCI)) and chromatographic separation, are listed.

Site	Sediment core/depth	Environmental setting	iGDGT-0 Peak area (arb. u.)	ext-iGDGT-0 Peak area (arb. u.)	di-ext-iGDGT Peak area (arb. u.)	Extended vs regular iGDGT-0 %	Ionization mode	LC-separation method	Measurement date	Cruise/Sampling date
Guaymas Basin	4568-1_12cm	oil-impregnated, hydrothermal, hot sediment	1005990	1722	n.d.	0.17	APCI	normal-phase (Becker et al., 2013)	03.02.2013	RV Atlantis cruise AT15-56, 2009
Guaymas Basin	4484_6B_20-22cm	hot, hydrothermal sediment (Mat Mound)	6408343	38875	n.d.	0.61	ESI	reversed-phase (Wörmer et al., 2013)	07.10.2025	RV Atlantis cruise AT15-40, 2008
Guaymas Basin	4484_6B_14-16cm	hot, hydrothermal sediment (Mat Mound)	14642435	107333	n.d.	0.73	ESI	reversed-phase (Wörmer et al., 2013)	07.10.2025	RV Atlantis cruise AT15-40, 2008
Guaymas Basin	4484_6B_10-12cm	hot, hydrothermal sediment (Mat Mound)	14389740	69681	n.d.	0.48	ESI	reversed-phase (Wörmer et al., 2013)	07.10.2025	RV Atlantis cruise AT15-40, 2008
Guaymas Basin	4484_6B_6-8cm	hot, hydrothermal sediment (Mat Mound)	9998597	23102	n.d.	0.23	ESI	reversed-phase (Wörmer et al., 2013)	07.10.2025	RV Atlantis cruise AT15-40, 2008
Guaymas Basin	4484_6B_0-2cm	hot, hydrothermal sediment (Mat Mound)	17750026	11481	n.d.	0.06	ESI	reversed-phase (Wörmer et al., 2013)	07.10.2025	RV Atlantis cruise AT15-40, 2008
White Oak River Basin	WOR 4-6cm	anoxic, estuarine sediment	1231649	n.d.	1059	0.09	APCI	normal-phase (Becker et al., 2013)	22.01.2016	2010
White Oak River Basin	WOR 12-14cm	anoxic, estuarine sediment	1625887	n.d.	1390	0.09	APCI	normal-phase (Becker et al., 2013)	22.01.2016	2010
White Oak River Basin	WOR 14-16cm	anoxic, estuarine sediment	1325301	n.d.	1298	0.10	APCI	normal-phase (Becker et al., 2013)	22.01.2016	2010
